# Utility of artificial intelligence-based conversation voice analysis for detecting cognitive decline

**DOI:** 10.1371/journal.pone.0325177

**Published:** 2025-06-02

**Authors:** Takeshi Kuroda, Kenjiro Ono, Masaki Onishi, Kouzou Murakami, Daiki Shoji, Shota Kosuge, Atsushi Ishida, Sotaro Hieda, Masato Takahashi, Hisashi Nakashima, Yoshinori Ito, Hidetomo Murakami

**Affiliations:** 1 Department of Neurology, Showa University School of Medicine, Tokyo, Japan; 2 Department of Neurology, Kanazawa University Graduate School of Medical Sciences, Kanazawa, Japan; 3 ExaWizards Inc., Tokyo, Japan; 4 Department of Radiology, Showa University School of Medicine, Tokyo, Japan; 5 Department of Neurology, Kawasaki Memorial Hospital, Kanagawa, Japan; Clinical Investigation Center, TUNISIA

## Abstract

Recent developments in artificial intelligence (AI) have introduced new technologies that can aid in detecting cognitive decline. This study developed a voice-based AI model that screens for cognitive decline using only a short conversational voice sample. The process involved collecting voice samples, applying machine learning (ML), and confirming accuracy through test data. The AI model extracts multiple voice features from the collected voice data to detect potential signs of cognitive impairment. Data labeling for ML was based on Mini-Mental State Examination scores: scores of 23 or lower were labeled as “cognitively declined (CD),” while scores above 24 were labeled as “cognitively normal (CN).” A fully coupled neural network architecture was employed for deep learning, using voice samples from 263 patients. Twenty voice samples, each comprising a one-minute conversation, were used for accuracy evaluation. The developed AI model achieved an accuracy of 0.950 in discriminating between CD and CN individuals, with a sensitivity of 0.875, specificity of 1.000, and an average area under the curve of 0.990. This voice AI model shows promise as a cognitive screening tool accessible via mobile devices, requiring no specialized environments or equipment, and can help detect CD, offering individuals the opportunity to seek medical attention.

## Introduction

The number of people with dementia is increasing worldwide [1]. Although prevention, treatment, and care through early detection are possible, dementia often remains unrecognized or undetected for a long time. Thus, a cost-effective method is required to detect early cognitive decline (CD) and dementia. Recently, the practical application of disease-modifying therapies (DMTs) for Alzheimer’s disease (AD) has been proposed. For example, lecanemab has been shown to reduce amyloid-β protein in early AD, resulting in a moderately slower decline in cognition and function compared to placebo [2]. To maximize the benefit of DMT, early medical consultation and diagnosis are essential for patients with CD. Despite the existence of nationwide dementia screening programs aimed at early detection, participation rates in Japan remain low. Accordingly, the number of cases where DMT could be beneficial but is not applied is expected to increase due to delays in detecting and diagnosing CD. While brain scans and body fluid biomarkers can detect the early stages of dementia, they are either invasive or expensive for screening purposes [3]. Therefore, a simple, non-invasive screening test for cognitive function that can be performed outside healthcare facilities is needed to encourage patients to seek medical attention.

Dementia is categorized as a neurocognitive disorder in the Diagnostic and Statistical Manual of Mental Disorders (DSM-5). It encompasses a range of disorders characterized by cognitive impairments in areas such as attention, planning, inhibition, learning, memory, language, visual perception, spatial skills, and social skills [4]. In particular, language abilities are often impaired in the early stages of dementia, with symptoms such as aphasia, pauses, reduced vocabulary, and other language-related deficits [5]. AD, dementia with Lewy bodies (DLB), and vascular dementia (VaD) are the most common types of dementia worldwide. Previous studies have reported changes in syntactic complexity, lexical content, speech production, fluency, and semantic content during the early stages of AD, with language ability shown to correlate with overall cognitive function [6,7]. Patients with DLB exhibit reduced speech fluency, characterized by a slower overall speech rate and long pauses between sentences [8]. Language disturbances in VaD are similar to those in AD, with impairments in semantically mediated language tasks [9]. Thus, language is a suitable cognitive function for assessing CD in the early stages of dementia.

Recent advancements in artificial intelligence (AI) have highlighted its potential in revolutionizing dementia diagnosis and care. Agbavor and Liang demonstrated that large language models (LLMs), such as GPT-3, can predict dementia with high accuracy by analyzing spontaneous speech [10]. Their approach outperformed traditional acoustic feature-based methods and showed promise for early diagnosis through simple, non-invasive speech analysis. Similarly, Treder et al. emphasized the transformative potential of LLMs in dementia care and research, including their use in enhancing cognitive assessments and providing personalized interventions via accessible digital platforms [11]. Additionally, Agbavor and Liang proposed an end-to-end AI model utilizing Data2Vec, a self-supervised algorithm, for AD detection and severity prediction based on voice data [12]. This model offers a cost-effective and scalable solution for community-based AD screening. Building on these advancements, AI-based technologies now facilitate the development of efficient and accessible methods for early dementia detection.

AI is expected to improve screening performance by extracting more features from a single test with fewer errors resulting from subjective judgments [13]. In addition, analyzing large datasets allows AI-based digital biomarkers to capture more features, improving accuracy and enabling more objective inferences compared to manual analysis [14]. AI-based cognitive function assessments include computerized cognitive tests [15,16], computer-assisted interpretation of brain scan images [17], observation and evaluation of gait, hand, and eye movements [18–20], as well as speech, conversation, and language tests [21–23]. However, existing AI-based cognitive assessment methods often require specific environments and equipment, and none have yet become routine in clinical practice. Cognitive screening tools used outside medical institutions should be quick, convenient, and usable anywhere. Using conversational voices presents a simple and useful tool for cognitive screening because it does not rely on specialized environments or equipment. In recent years, the usefulness of high-level phonetic feature models, such as hidden unit bidirectional encoder representations from transformers (HuBERT), has been demonstrated in dementia detection [24,25]. We focused on how phonetic features in daily conversations reflect CD and aimed to develop a machine learning (ML)-based voice AI to detect CD from one-minute conversations.

## Methods

### Research outline

**Fig 1:**
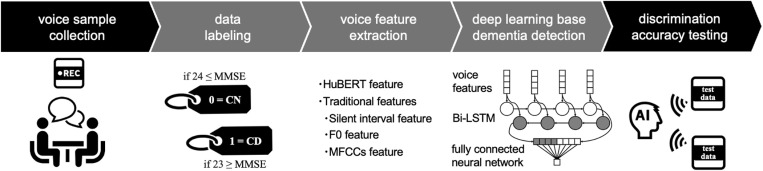
Overview of Voice Sample Collection and Utilization. The collected voice samples and data labels were used for ML. The main ML procedures included voice feature extraction and deep learning. The accuracy of the model was confirmed using an ML-based voice AI system. ML: machine learning; MMSE: Mini-Mental State Examination; CN: cognitively normal; CD: cognitively declined; HuBERT: Hidden Unit BERT; MFCC: Mel-frequency cepstral coefficients.

This study aimed to develop an ML-based voice AI capable of detecting CD from short conversational voice samples. The process involved five key steps: 1) collecting voice samples, 2) labeling the collected voice data, 3) voice feature extraction, 4) applying features to the deep-learning model, and 5) confirming the accuracy of the developed voice AI model using test voice data (Fig 1). The study was approved by the Ethics Committee of Showa University School of Medicine (approval number: 21–018-B) and was conducted in accordance with the principles of the Declaration of Helsinki (as revised in 2013).

### Voice sample collection

Since no large-scale Japanese voice dataset for dementia detection exists, we created our own dataset. We enrolled consecutive patients aged 60 and older who consulted the Memory Clinic of the Department of Neurology, Showa University School of Medicine, Japan, for concerns related to memory loss between January 2022 and September 2023. All participants were of Japanese origin, and voice samples were collected in standard Japanese. The voice recordings were gathered during conversations while the participants engaged in original tasks and underwent neuropsychological assessments, including the Mini-Mental State Examination (MMSE), Hasegawa’s Dementia Scale-Revised (HDS-R), and the Montreal Cognitive Assessment (MoCA) [26–28]. The original tasks consisted of the following: 1) conversational speech about “something fun you experienced recently;” 2) responses to three meal-related questions: “What did you eat today?,” “Please describe the contents of your meals yesterday, starting with breakfast,” and “What was the most memorable meal?;” and 3) a picture description task using The Cookie Theft Picture [29]. All psychological tests and tasks were conducted face-to-face between the examiner (a psychologist or neurologist) and the participant in a quiet room with a noise level between 40 and 50 dB. Voice recordings were made using a 6^th^-generation iPad equipped with a microphone placed on a table between the examiner and the participant. Participants were informed that their conversations would be recorded during the examination. The recorded voice samples were stored on the iPad until the ML phase. Written informed consent was obtained from all participants.

### Data labeling

The MMSE is one of the most widely employed tests for cognitive screening [26]. With a cutoff score set at 23/24, the combined sensitivity and specificity for detecting dementia have been reported as 0.81 and 0.89, respectively [30]. Voice data associated with MMSE scores of 23 or lower were labeled as “1” = CD, whereas those with MMSE scores of 24 or higher were labeled as “0” = cognitively normal (CN).

### Voice feature extraction

We used a comprehensive approach, utilizing multiple voice features to detect potential signs of cognitive impairment. Through preprocessing methods and feature extraction models, our goal was to maximize the accuracy of the dementia detection system by implementing a neural network ML pipeline.

#### Preprocessing.

The preprocessing step involved standardizing and preparing the audio data for feature extraction. First, all audio files were converted to a consistent format with a 16-bit, 16,000 Hz mono waveform, ensuring uniformity across the dataset. Noise reduction techniques were applied using the voice enhancement model from ESPnet2 (https://github.com/espnet/espnet) to minimize background noise. Second, we preprocessed the training set of voice data using Pyannote-audio (https://github.com/pyannote/pyannote-audio), an open-source Python toolkit for speaker diarization, to separate the voices of the examinee and examiner. The test set was manually processed in the same format. Finally, the audio signals were normalized to ensure that variations in volume did not affect the feature extraction process.

#### HuBERT feature.

We used the HuBERT model to extract deep speech representations from the audio data. Unlike approaches such as those based on large language models that rely on linguistic features derived from transcription, our method focuses exclusively on acoustic features. To achieve this, we adopted HuBERT, a self-supervised model pre-trained on large-scale, unlabeled speech data, designed to capture both phonetic and prosodic information [24]. We used a pre-trained Japanese HuBERT model, provided by Rinna Co., Ltd. (https://huggingface.co/rinna/japanese-hubert-base), to extract features, leveraging its ability to encode both short-term and long-term dependencies in speech. The output from the last layers of HuBERT was used as high-level representations, serving as input to the subsequent dementia detection model.

#### Traditional acoustic features.

In addition to the HuBERT features, we extracted several traditional acoustic features commonly used in speech processing for dementia detection, using the librosa Python package for music and audio analysis (https://librosa.org/doc/latest/index.html). Silent interval features, which are indicative of CD, were extracted using the voiced_probs output from librosa.pyin. This probability differentiates between voiced and unvoiced segments of the audio signal, and we used these values directly as input for the next step. Fundamental frequency (F0) features were computed using librosa.pyin, which estimates pitch values from the audio signal. These values capture prosodic variations, such as pitch range, average pitch, and pitch stability, all of which are relevant for detecting speech irregularities associated with dementia. Mel-frequency cepstral coefficients (MFCCs) were extracted using librosa.feature.mfcc with an n_mfcc value of 20. For each audio segment, the maximum, mean, and delta values of the MFCCs were calculated and concatenated for the next input stage, allowing the capture of both static and dynamic spectral characteristics. These features offer valuable insights into the speaker’s articulation and vocal quality.

### Deep learning-based dementia detection

The extracted features (HuBERT features, silent interval features, F0 features, and MFCCs) were used as input for an ML model designed to detect signs of dementia in speech. For each 60-second audio sample, feature extraction was performed every five seconds, with a one-second overlap between consecutive segments. The extracted features included HuBERT features (768 dimensions), silent interval features (100 dimensions), F0 features (100 dimensions), and MFCCs (60 dimensions). These features were then fed into a fully connected (FC) layer. The structure of the FC layer consisted of two layers, each with 1128 and 768 neurons, respectively. Each layer utilized ReLU activation functions. Following the FC layer, the time-step ordered features were fed into a bidirectional long short-term memory (Bi-LSTM) network, which was designed to capture temporal dependencies in the data. The Bi-LSTM network consisted of two layers with 512 hidden units each. After processing through the Bi-LSTM, the final hidden state was passed through two FC layers with 1024 and 512 neurons to perform the classification. Details of the hyperparameters and the neural network architecture are provided in the S1 and S2 Tables. The system was trained on a labeled dataset, where speech samples were labeled as “0” or “1” according to MMSE scores, with “0” representing CN individuals and “1” indicating CD. The final system was capable of automatically classifying speech samples as either dementia-positive or dementia-negative based on the extracted features.

### Discrimination accuracy testing

Twenty voice samples were prepared to assess the discrimination accuracy of the ML-based voice AI model. The test data consisted of one-minute conversations about “something fun you experienced recently,” a segment from the original task. We chose this question to encourage patients to recall and describe personal episodic memories, which are more likely to elicit natural and emotionally rich open-ended responses compared to one-answer questions such as “what did you eat for lunch?” or “what is your favorite movie?”. Additionally, since the task involves approximately one minute of conversation, we aimed to provide a topic broad enough to allow for elaboration. None of the voice data used for testing were included in the model training. The CN test data encompassed individuals diagnosed with subjective CD (SCD) and mild cognitive impairment (MCI), whereas the CD test data consisted of individuals diagnosed with AD, DLB, and VaD, the three major types of dementia. The ML-based voice AI model outputs a probability (ranging from 0 to 1) indicating the likelihood that the voice belongs to a CD individual. A probability value of 0.5 or higher was set as the threshold for diagnosing CD.

### Clinical diagnosis

All patients underwent a detailed interview, neurological examination by an experienced neurologist, blood tests, clinical dementia rating (CDR), MMSE, and brain MRI. Additional examinations were performed as necessary for clinical diagnosis. SCD was characterized by self-reported memory complaints, a CDR score of 0, MMSE scores within the normal range for cognition (MMSE ≥ 28), and no evidence of impairment in functional activities. The diagnosis of MCI was based on the criteria proposed by Petersen [31], which included a memory complaint corroborated by an informant, a global CDR score of 0.5, and a cognitive decline indicated by an MMSE score between 24 and 27, but with no evidence of functional impairment as revealed by the clinical interview. Diagnoses were made according to established guidelines for each condition. Alzheimer’s disease (AD) diagnoses followed the guidelines of the National Institute on Aging–Alzheimer’s Association workgroups [32]. Vascular cognitive disorders were diagnosed using the criteria established by the International Society for Vascular Behavioral and Cognitive Disorders [33]. The revised criteria for the clinical diagnosis of dementia with Lewy bodies (DLB) were applied [34], while the revised diagnostic criteria for the behavioral variant of frontotemporal dementia (FTD) were used for FTD cases [35]. Corticobasal degeneration (CBD) was diagnosed based on clinical criteria [36], and Parkinson’s disease (PD) was diagnosed using the International Parkinson and Movement Disorder Society criteria [37]. Lastly, idiopathic normal pressure hydrocephalus (iNPH) diagnoses followed the third edition of the Japanese Guidelines for Management of iNPH [38].

### Statistics

An unpaired t-test was employed to analyze the differences in mean age, years of education, MMSE scores, and CDR scores between the CD and CN groups for voice samples used in model training and testing to confirm accuracy. A chi-square test was conducted to examine the male-to-female ratio of the samples. All tests were two-tailed and conducted using SPSS version 29.0.1.0 (IBM Corp., Armonk, NY, United States). Statistical significance was defined as an adjusted *p-*value of less than 0.05. The results are presented as mean and standard deviation (SD).

## Results

### Voice datasets for machine learning

**Fig 2:**
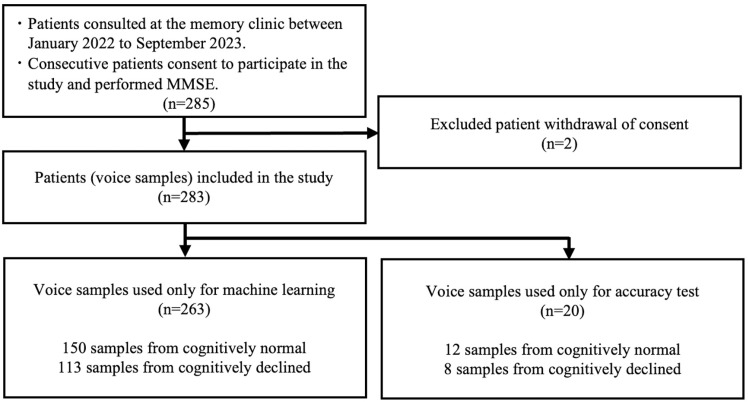
Study Protocol. MMSE: Mini-Mental State Examination.

Voice samples were collected from 285 consecutive patients who visited the Memory Clinic, with their consent to participate in the study. However, two patients withdrew their consent, leaving voice samples from 283 patients to be used in this study. For the accuracy confirmation test, 20 voice samples were selected, leaving 263 voice samples (155 females) for model training (Fig 2). The clinical diagnoses of the 263 patients included AD (n = 85, 32.3%), MCI (n = 78, 29.7%), SCD (n = 34, 12.9%), VaD (n = 17, 6.5%), DLB (n = 12, 4.6%), PD (n = 9, 3.4%), iNPH (n = 7, 2.7%), brain tumor (n = 4, 1.5%), multiple system atrophy (n = 3, 1.1%), depression (n = 3, 1.1%), CBD (n = 2, 0.8%), FTD (n = 2, 0.8%), and neuronal intranuclear inclusion disease (n = 1, 0.4%). Clinical diagnosis was not feasible for six patients (2.3%) owing to inadequate testing. Among the 263 samples, 113 samples (74 females) were categorized as CD (MMSE scores of 23 or lower). The remaining 150 voice samples were categorized as CN (MMSE scores of 24 or higher). A summary of the voice samples used for ML is given in Table 1.

**Table 1:** Summary of Voice Dataset Used for ML.

	All	CN (MMSE ≥ 24)	CD (MMSE ≤ 23)	*p* – value
Data labeling		0	1	
n (% female)	263 (58.9)	150 (54.0)	113 (65.5)	0.06
MMSE (mean ± SD)	23.5 ± 5.0	27.1 ± 4.1	18.7 ± 5.1	6.51 × 10^-54
Age (mean ± SD)	77.8 ± 9.4	75.1 ± 10.4	81.4 ± 7.2	5.00 × 10^-9
Educational years (mean ± SD)	13.3 ± 2.6	13.9 ± 2.5	12.5 ± 2.4	1.58 × 10^-5
CDR (mean ± SD)	0.8 ± 0.6	0.5 ± 0.3	1.2 ± 0.5	3.70 × 10^-25

MMSE: Mini-Mental State Examination; CDR: clinical dementia rating; CN: cognitively normal; CD: cognitively declined; SD: standard deviation.

### Discrimination accuracy of ML-based voice AI model

**Fig 3:**
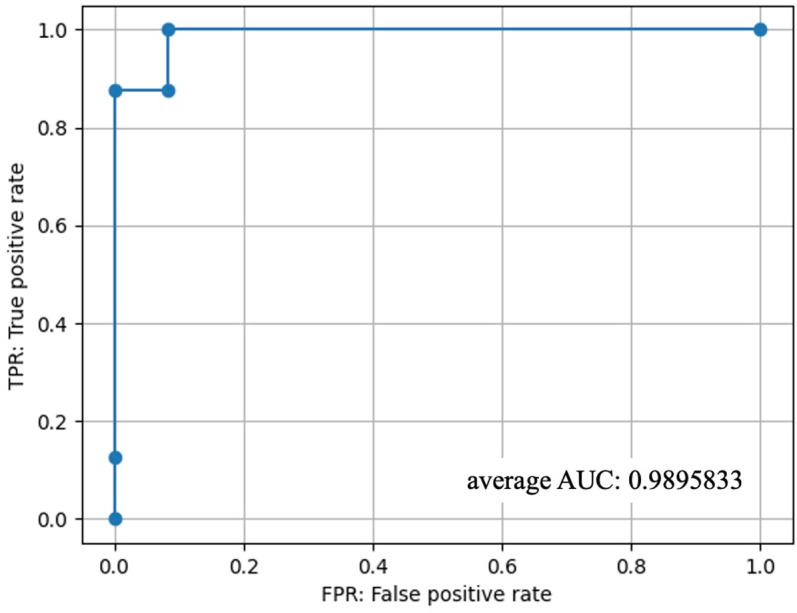
Receiver Operating Characteristic Curve of ML-based Voice AI Model.

The discrimination test used 20 voice datasets, comprising eight CD samples (four females, mean age 77.5 ± 9.8 years, mean education years 13.0 ± 2.7, mean MMSE score 18.4 ± 4.0, mean CDR 1.5 ± 0.5) and 12 CN samples (seven females, mean age 75.0 ± 9.5 years, mean education years 14.2 ± 2.0, mean MMSE score 26.8 ± 2.1, mean CDR 0.3 ± 0.3). No significant differences were observed in the percentage of females (*p* = 0.71), age (*p* = 0.56), or years of education (*p* = 0.16) between the CD and CN groups. However, patients with CD exhibited significantly lower MMSE scores (*p* = 0.0003) and higher CDR scores (*p* = 0.0002). The clinical diagnoses for the CN group included five cases of SCD and seven cases of MCI, while the CD group comprised five cases of AD, two cases of DLB, and one case of VaD (Table 2). The distribution of AD, DLB, and VaD cases in the CD group was determined to reflect the ratio of real-world prevalence. Following ML, the voice AI model was able to discriminate between CD and CN with an accuracy of 0.950, a sensitivity of 0.875 (probability of correctly identifying a CD as a CD), and a specificity of 1.000 (probability of correctly identifying a CN as a CN). The average area under the curve was 0.990 (Fig 3).

**Table 2:** Result of discrimination test using speech AI model after ML.

Test samples				Discrimination result		
No.	Clinical diagnosis	MMSE	Labeling	Probability	Decision	Result
1	SCD	30	CN (0)	0.0367	CN	correct
2	SCD	29	CN (0)	0.2156	CN	correct
3	SCD	29	CN (0)	0.1927	CN	correct
4	SCD	28	CN (0)	0.2509	CN	correct
5	SCD	28	CN (0)	0.2191	CN	correct
6	MCI	27	CN (0)	0.2174	CN	correct
7	MCI	26	CN (0)	0.2940	CN	correct
8	MCI	25	CN (0)	0.2372	CN	correct
9	MCI	25	CN (0)	0.1882	CN	correct
10	MCI	25	CN (0)	0.3585	CN	correct
11	MCI	24	CN (0)	0.4930	CN	correct
12	MCI	24	CN (0)	0.3877	CN	correct
13	AD	23	CD (1)	0.4734	CN	incorrect
14	AD	23	CD (1)	0.5320	CD	correct
15	AD	21	CD (1)	0.7185	CD	correct
16	DLB	19	CD (1)	0.7641	CD	correct
17	AD	18	CD (1)	0.5831	CD	correct
18	VaD	17	CD (1)	0.7356	CD	correct
19	AD	13	CD (1)	0.7136	CD	correct
20	DLB	13	CD (1)	0.8704	CD	correct

MMSE: Mini-Mental State Examination; CN: cognitively normal; CD: cognitively declined; SCD: subjective cognitive decline; MCI: mild cognitive impairment; AD: Alzheimer’s disease; DLB: dementia with Lewy bodies; VaD: vascular dementia.

## Discussion

This voice AI model is novel in its ability to discriminate between CD and CN individuals with high accuracy by analyzing one-minute conversation samples. The model’s high discrimination accuracy of 0.950, attained through a simple method, demonstrates the feasibility of using short conversational voice samples as a practical screening tool for analyzing cognitive function and alerting individuals to possible CD.

Extensive research on AI-based dementia assessment, particularly for AD, has been conducted worldwide. Practical digital biomarkers for diagnosing dementia can reduce the burden on clinical practice. Conversation and language abilities are impaired in the early stages of most types of dementia [8]. Recent studies have focused on AI-based assessments that employ speech and language. Typical testing procedures involve extracting relevant features and inputting them into machine- or deep-learning classifiers to identify patterns consistent with dementia. Two primary types of features—acoustic and linguistic—can be extracted and analyzed from human conversational voice [13]. Acoustic features describe how individuals articulate speech, while linguistic features pertain to the content, such as vocabulary, grammar, and syntax. According to a recent review, the extraction and analysis of linguistic features achieved better accuracy (0.925) than the utilization of acoustic features alone (0.786). Combining linguistic and acoustic features in AI analysis outperformed both, with an accuracy of 0.939 [13]. In contrast, the voice analysis AI developed in this study predominantly analyzed acoustic features and achieved a higher accuracy rate (0.950) than previous studies that used acoustic features alone [13]. The use of acoustic features alone may offer certain advantages over linguistic features: conversion errors are avoided because transcribing conversational speech into text is not required, and only a short conversation sample is needed for analysis.

Recently, two main types of tests have been developed to analyze conversational voices: the picture description test (where participants describe a picture while their voice is recorded) and interview-based conversations. In our study, although the voice data used for ML were obtained from both picture descriptions and interviews, the discrimination test was based only on one-minute conversations. This conversation was not provoked by a specific task, as in the picture description test, but was rather a spontaneous conversation based on an individual’s episodic memory, resembling an interview task. In interview-based diagnoses, subjects respond to multiple questions posed by humans or avatars, and their acoustic and linguistic features are analyzed to identify patients with CD or dementia [23,39,40]. However, these tests require subjects to answer multiple questions, making them time-consuming and potentially giving the impression that the subject is being tested for cognitive function. In contrast, our method of discriminating between CD and CN individuals using a short conversational voice sample obtained from just one question offers a simpler approach that could be widely used in clinical settings. Another advantage is that, unlike tasks with fixed correct answers, freeform conversations allow for open-ended answers, reducing the learning effect and making repeated administration easier. The proposed voice AI model identified CN individuals with 100% accuracy. The absence of false positives (that is, diagnosing a CN individual as CD) indicates the usefulness of this method as a screening tool in real clinical situations, helping to prevent unnecessary worry or anxiety in healthy individuals and avoiding the additional medical burden and costs associated with further testing.

However, this process has several limitations. The voice AI model developed in this study is capable of detecting CD. However, as dementia is defined as “a state of cognitive decline that interferes with daily and social life,” our voice AI model, which does not account for patients’ living contexts, is currently unable to determine whether an individual meets the clinical criteria for “dementia.” This study primarily focused on assessing the simple cognitive evaluation capabilities of the voice AI model. We plan to extend our work by incorporating machine learning approaches that analyze not only MMSE scores but also data from other neuropsychological assessments, patients’ daily and social life contexts, and biomarkers associated with dementia. In addition, we believe that regression analysis using numerical indicators such as the MMSE may be a particularly valuable tool in the clinical setting for assessing the severity of cognitive impairment in patients. We intend to consider this approach as a potential area for future research. It is essential to recognize that AI is not a substitute for human expertise but rather a powerful tool that enhances decision-making and supports accurate diagnosis. Determining whether the voice AI model can surpass clinicians’ diagnostic capabilities for dementia remains a critical area for future research. Additionally, our voice AI model cannot detect mild CD, as our MMSE cutoff score was set at 23/24. Identifying patients with cognitive impairment before progression to dementia, specifically at the MCI levels is crucial, as early intervention provides more opportunities for prevention, care, and effective treatment, such as DMT for AD. To identify cognitive impairment at the MCI level, it is necessary to adjust the MMSE cutoff score for data labeling and incorporate CDR results of 0.5, along with more appropriate neuropsychological tests, into the ML process. Although the MMSE is a widely used and simple neuropsychological test employed globally, and it demonstrates high sensitivity for moderate to severe cognitive impairment, it has the drawback of low sensitivity for detecting MCI [41]. Additionally, the MMSE includes relatively simple items for assessing language, which limits its utility in identifying mild language impairments. One of the psychological tests useful for detecting MCI is the Montreal Cognitive Assessment (MoCA). Using a cutoff score of 26, the MMSE showed a sensitivity of 18% for detecting MCI, whereas the MoCA identified 90% of individuals with MCI [28]. The MoCA is also a simple cognitive screening tool with high sensitivity and specificity for detecting currently conceptualized MCI, even in patients who fall within the normal range on the MMSE. Future research should include additional ML using voice samples from individuals with milder CD to explore the potential for detecting such conditions with higher accuracy.

Another limitation is that voice samples were collected only once per individual, making it challenging to evaluate the consistency of the AI model’s decisions for the same individual over time. This raises the possibility that discrimination results could vary depending on daily conditions, such as lack of sleep, alcohol consumption, or temporary forgetfulness, which could lead to incorrect decisions. Additionally, as this study was conducted on patients attending the Memory Clinic at our hospital, recruiting cognitively impaired individuals under the age of 60 was challenging. As voice samples were exclusively collected from individuals aged 60 years and older, our voice AI model currently lacks the capability to detect early-onset dementia in younger populations. Obtaining voice samples from younger individuals would indeed be feasible, and we recognize the value of extending our research to include this population. We plan to explore the inclusion of younger age groups in future studies to address this limitation and enhance the model’s generalizability. Moreover, we used only standard Japanese voice samples for ML, and while Japanese dialects exist, the model’s effectiveness with dialects remains unknown. To solve these problems, a more robust model could be developed by adding voice samples from younger individuals or those speaking in dialects, potentially sourced from TV or web content, for pre-training the HuBERT model.

In recent years, significant progress has been made in developing AI systems that use observational methods by integrating multiple assessments to assess the impact of CD on daily life in the elderly. While it remains challenging to accurately distinguish CD individuals using only simple evaluation methods, our voice AI model enables the development of AI-based medical software for detecting CD using minute-long conversations, accessible via mobile devices such as smartphones. Digital biomarkers based on language can also be used for detecting mental disorders such as depression [42]. We believe that an AI-assisted, simple cognitive function screening tool, using short conversational voice samples, can be valuable in detecting CD and encouraging people to seek medical attention. In this study, as an initial experiment, we validated the model’s accuracy using the holdout method with fixed samples. To enhance its applicability for real-world implementation, we plan to incorporate cross-validation in future evaluations.

## Supporting information

S1 Table Neural Network Architecture. (DOCX)

S2 Table Hyperparameters. (DOCX)

S3 Data Datasets Used for Machine Learning and Discrimination Test. (XLSX)
