# Supplementary material for: Utility of artificial intelligence-based conversation voice analysis for detecting cognitive decline
Source: PLoS One. 2025 Jun 2;20(6):e0325177. doi: 10.1371/journal.pone.0325177 (PMC12129157; doi:10.1371/journal.pone.0325177)
Supplement: S1 Table — (DOCX) [file pone.0325177.s001.docx]

| **No** | **Type** | **Input Shape** | **Output Shape** | **Activation** |
| --- | --- | --- | --- | --- |
| 1 | Fully Connected | (1128) | (768) | ReLU |
| 2 | Fully Connected | (768) | (512) | ReLU |
| 3 | Bi-LSTM | (512, 15) | (1024, 15) | None |
| 4 | Fully Connected | (1024) | (512) | ReLU |
| 5 | Fully Connected | (512) | (1) | Sigmoid |
