# Supplementary material for: Utility of artificial intelligence-based conversation voice analysis for detecting cognitive decline
Source: PLoS One. 2025 Jun 2;20(6):e0325177. doi: 10.1371/journal.pone.0325177 (PMC12129157; doi:10.1371/journal.pone.0325177)
Supplement: S2 Table — (DOCX) [file pone.0325177.s002.docx]

| **Parameter** | **Value** |
| --- | --- |
| Optimizer | Adam |
| Learning late | 0.0001 |
| Loss function | Binary Cross Entropy |
| Batch size | 8 |
| Epochs | 56 |
